# Supplementary material for: Content-rich biological network constructed by mining PubMed abstracts
Source: BMC Bioinformatics. 2004 Oct 8;5:147. doi: 10.1186/1471-2105-5-147 (PMC528731; doi:10.1186/1471-2105-5-147)
Supplement: Additional File 2 — The original results of the above study (non-essential files are deleted to keep the file size under the limit set by BMC bioinformatics). [file 1471-2105-5-147-S2.bz2 › chilibotAdditionalFile2/dip05/628ID11089964E11015/html/SRP9_SRP14.html]

 


 **SRP9** and **SRP14** 
  
Found 21 abstracts in PubMed, retrieved 05.  
 

 What does Google say? 
 PDF only 
| .edu only 

---

**Interactive relationship** (e.g. stimulation, inhibition, etc)

**Neutral relationship**- The proteins  **SRP9**  and  **SRP14**  first heterodimerize and then initially bind to the Alu RNA 5 domain.  Ref: 11350037 RNA, 2001
- This elongation arrest activity requires the  **SRP9**  14 subunit of the particle and interactions of the C terminus of  **SRP14** .  Ref: 10921896 EMBO J, 2000

**Non-interactive relationship** (e.g. studied together, co-existance, homology, etc.)

- This release includes 11 new SRP RNA sequences total of 129, two protein  **SRP9**  sequences total of seven, two protein  **SRP14**  sequences total of 10, two protein SRP19 sequences total of 1 0 new SRP54 ffh sequences total of 66, two protein SRP68 sequences total of seven and two protein SRP72 sequences total of nine.  Ref: 11125080 Nucleic Acids Res, 2001
- The Alu domain of the mammalian signal recognition particle SRP comprises the heterodimer of proteins  **SRP9**  and  **SRP14**  bound to the 5 and 3 terminal sequences of SRP RNA.  Ref: 11089964 Nature, 2000
